# Early detection of antiseizure medication inefficacy using an implantable continuous EEG system and a personalized model: a case study

**DOI:** 10.1016/j.ebr.2025.100829

**Published:** 2025-09-23

**Authors:** A. Reynolds, A. Lai, D.B. Grayden, M.J. Cook, A. Peterson

**Affiliations:** a Department of Biomedical Engineering, The University of Melbourne, Melbourne, Australia; b Department of Medicine, The University of Melbourne, Melbourne, Australia; c Department of Neurosciences, St. Vincent’s Hospital, The University of Melbourne, Melbourne, Australia; d Graeme Clark Institute, The University of Melbourne, Melbourne, Australia; e Epi-minder Pty Ltd, Melbourne, Australia

**Keywords:** Epilepsy, Anti-epileptic drug, Efficacy, Biomarker, Sub-scalp, Cycles

## Abstract

Evaluating anti-seizure medication (ASM) efficacy can be limited by inaccurate seizure diaries and periodic fluctuations in seizure frequency, known as seizure cycles. These limitations may prolong monitoring of ineffective treatments. This study explores implantable continuous EEG monitoring (iCEM™) with Epiminder’s Minder® system and timeseries modelling to improve efficacy assessments.

This retrospective case study examines a 49-year-old female with drug-resistant focal epilepsy with iCEM (Nov. 2019). The participant maintained a seizure diary and was followed-up for 3 years. ASMs were changed in Aug.-Oct. 2020. A personalised autoregressive model incorporating interictal epileptiform discharge cycles to project 3-monthly seizure rates was trained and validated on post-drug data then tested on four held-out datasets (two pre-drug and two post-drug). The Kruskal-Wallis test assessed model performance between drug periods (α = 0.05).

Only 37 % of seizures were reported. Post-drug, diary-reported seizures increased while detected seizures decreased, but both remained within normal seizure rate variability. ASM inefficacy was addressed after 3 years. The autoregressive model mean squared error post-drug was 0.17 and 0.13 seizures per day over 3-months^2^, which were significantly different from pre-drug (0.49 and 0.58 seizures per day over 3-months^2^, H=336.82, p = 2.44exp.^-89^), suggesting the model could recognise when an altered drug regimen affected seizure rate and interictal epileptiform discharges.

Seizures identified using iCEM combined with an individualised model may be able to distinguish drug-induced changes in seizure rate from normal variability. This proof-of-concept study offers useful information towards the development of methods that can support early treatment assessments, potentially shortening the time to find an optimal therapy.

## Highlights

• Distinguishing drug effects from natural seizure rate variability is challenging.
• Implanted EEG with a personalised model may detect drug effects on seizure rate.
• This novel method may facilitate early evaluation of antiseizure medications.
• Further research is needed to optimise and evaluate this method for clinical use.

## Graphical abstract

**Figure:**
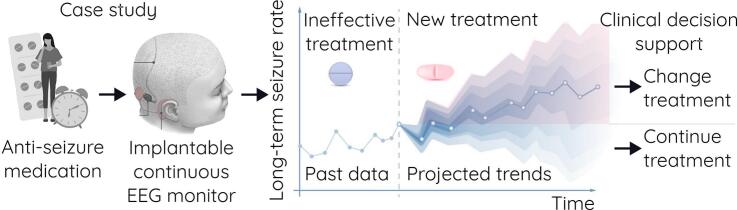

## 1 Introduction

Assessing anti-seizure medication (ASM) efficacy in the treatment of epilepsy poses the extraordinarily challenging task of predicting the likelihood of achieving lifelong seizure freedom [1]. In clinical practice, a prognosis is instead determined by integrating information such as past seizure rates, consecutive seizure-free days, clinical factors, and statistical probabilities derived from research studies [2]. However, this approach can be limited by generalisability, inaccurate seizure diaries, and seizure cycles – periodic fluctuations in seizure-risk – which may cause natural changes in seizure rate to be misattributed to the effects of ASMs [[3], [4], [5], [6]].

Implantable continuous electroencephalography (EEG) monitoring (iCEM) could reduce biases associated with both diaries and cross-sectional assessments of cyclical seizure counts [7,8]. However, relying solely on seizure counts without statistical modelling means depending on past trends and clinical expertise to assess efficacy. While iCEM may estimate seizure rates more accurately than diaries [7,9], and influence ASM prescribing [10], its potential to shorten the time to identify an optimal treatment or improve clinical outcomes remains to be investigated. Moreover, additional benefits may arise from advanced analytical approaches capable of anticipating treatment earlier and more accurately than clinical judgement alone.

Statistical models using long-term (month-years) intracranial or sub-scalp EEG biomarkers have been studied for predictions related to epilepsy diagnosis, seizure detection, and seizure forecasting [[11], [12], [13]]. However, their use in projecting long-term trends in seizure rate for assessing ASM efficacy remains unexplored. Long-term seizure rate projections could be approached similarly to economic modelling, where financial trends are projected to plan budgets for future months or years. Therefore, timeseries models such as autoregressive models, might be useful for projecting future seizure rates. Autoregressive models use a linear combination of past observations, and are designed to handle longitudinal, high-frequency, and volatile timeseries [14]. These models have already been applied to short-term EEG for seizure detection [[15], [16], [17], [18]], surgical resection of the seizure-onset zone[19] and short-term seizure prediction [20,21].

This study aims to determine whether an autoregressive model incorporating long-term seizure rates and seizure cycles tracked by interictal epileptiform discharges (IEDs) can distinguish drug regimens. We hypothesise that a personalised drug-specific autoregressive model can perform well on data from the same drug regimen but will show changes in performance when medications are altered. With further research, such changes in model performance could help differentiate normal seizure rate variability from true treatment effects, potentially enabling early treatment evaluation to rapidly identify an optimal therapy.

## 2 Methodology

### 2.1 Study design, consent and ethics approvals

This proof-of-concept retrospective case study involves a participant from the prospective, 3-year-long first in-human trial of the Minder® iCEM system (ACTRN12619001587190) [9,22]. The iCEM is a sub-scalp EEG, continuously recording electrical activity from two channels over bilateral centroparietal regions of the brain. It is able to capture a range of seizure patterns including focal, bilateral focal and generalised [9].

The trial included two week-long video EEG monitoring sessions, using scalp electrodes placed according to the international 10–20 system (Compumedics® Siesta, Melbourne, Australia), conducted concurrently with iCEM recording. Due to clinical need, the participant changed chronic ASMs and was followed up to 3 years post-drug change.

Written informed consent was obtained, and the study was approved by St Vincent’s Hospital Melbourne Human Research Ethics Committee (HREC158/19).

### 2.2 Clinical outcomes

As per the International League Against Epilepsy, ASMs were efficacious if they achieved one year of seizure freedom or a seizure-free period three times the longest inter-seizure interval, whichever was longer [23]. Seizure severity was measured using the Liverpool Seizure Severity Scale 2.0. Quality of life was evaluated using the Quality of Life in Epilepsy Inventory (QOLIE-89). Questionnaires were scheduled for completion at 0, 4, 36, 72, 84, 96, 108, and 144 weeks after device implantation.

### 2.3 Event detection

Four-week seizure recall was obtained from the Liverpool Seizure Severity Scale 2.0. The participant and family members recorded seizures in a diary. Medications and missed doses were recorded in a medication log. EEG seizures and IEDs were detected by a convolutional neural network [22,24]. Given the large volume of EEG data collected over the three-year period, the proof-of-concept analysis was simplified to the presence or absence of a seizure, with seizure detections assigned a ≥ 99 % estimated probability of accuracy included. Gaps in the timeseries were addressed using linear interpolation with Gaussian white noise per Baud et al. (2018) [25] (see supplementary material).

### 2.4 Feature extraction

Seizure start times were converted into daily counts, with a causal 90-day moving average computed. IEDs per hour were analysed for cycles (1–45 days) using the continuous Morlet wavelet transform [25]. Statistically significant cycle periods were identified using a permutation test (false discovery rate of 0.95) [26]. Following Leguia et al. (2023) the sine and cosine phases were included in the autoregressive integrated moving average model with exogenous variables [26] (see supplementary material for details).

### 2.5 Division of data into training, validation and test sets

Due to the timing of the implantation of the iCEM system (November 2019), drug changes (August-October 2020), and two extended data gaps, the training and validation dataset was confined to 369 days between February 2021-February 2022. This occurred after the drug change. Test datasets were 3 – 4 months long, two occurred before the drug change (November 2019 − March 2020 and April − August 2020) and two after (October 2020 − January 2021 and April − July 2022).

### 2.6 Model selection, parameterisation and performance evaluation

Autoregressive (AR), moving average (MA), autoregressive integrated moving average models (ARIMA) with exogenous variables (ARIMAX) were used to project long-term seizure rates 14 days into the future (time horizon), updating daily. Input data was minimised to the longest lag duration plus one day, with hyperparameters (e.g., autoregressive lag, differencing, and moving average orders) optimised via grid search and repeated hold-out validation, using a 70/30 split iterated 100 times [27]. Performance was benchmarked against naïve models (AR(1) and MA(5)) using average mean squared error, with final models evaluated on the four held-out test datasets.

To ensure robustness, the final model was applied to surrogate timeseries, which were generated by random shuffling of the original timeseries, maintaining the train/validation/test splits for comparison.

The Kruskal-Wallis test with Wilcoxon post-hoc comparisons (α = 0.05 with Bonferroni correction) assessed performance differences. Models needed to show consistent performance on datasets from the same drug regimen but distinguish between pre- and post-drug periods. Significant differences in residual values indicated the model's ability to distinguish drug regimens.

## 3 Results

### 3.1 Clinical summary

A 49-year-old right-handed female with drug-resistant epilepsy due to bilateral periventricular nodular heterotopia participated in the safety trial (Nov 2019 − Nov 2022). The participant experienced focal seizures with impaired consciousness and focal to bilateral tonic-clonic seizures. Pre-drug video EEG monitoring captured only focal seizures; no focal to bilateral tonic–clonic seizures were observed. Significant underreporting was noted, with only 4 of 14 (28.47 %) seizures being reported, and presumed overreporting, with 4 events showing no clinical or EEG correlate. All video EEG seizures were detected by the iCEM.

During the trial, the median proportion of each day that the device was used was 83 % (IQR 100). The iCEM detected 3122 seizures, of which, 1169 (37.44 %) were recorded in the diary. Five reported seizures (0.16 % of all reported) were considered possible focal to bilateral tonic–clonic seizures; these were unprovoked and occurred prior to the drug change. The participant was uncertain about these five events, as they were unwitnessed, with no recollection and only postictal symptoms reported. At the end of the trial, the participant chose to retain the implant, indicating perceived or anticipated benefit.

Initially, the participant was prescribed lacosamide, pregabalin, and valproate. Valproate was subsequently replaced with brivaracetam following reports of unprovoked focal to bilateral tonic–clonic seizures and a focal seizure with impaired consciousness that resulted in a skin burn. The participant demonstrated excellent drug adherence, missing only one day in June 2021, requiring rescue clonazepam. No adverse drug effects were reported. The clinician observed an initial reduction in seizure frequency during drug titration, attributed to the new therapy, but the effect was short-lived and thought to be due to drug tolerance.

**Fig. 1:**
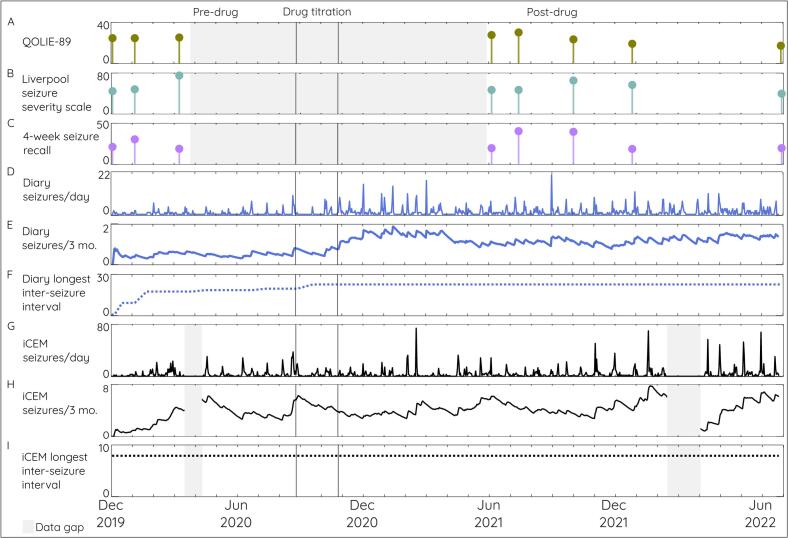
**Certainty in medication assessments is limited when relying on clinical experience combined with subjective or objective disease monitoring.** Changes in (A) quality of life, (B) seizure severity, and (C) 4-week seizure recall related to medication adjustments are difficult to interpret due to an extended gap between measurements, caused by COVID-19 lockdowns that prevented non-urgent clinical reviews. Discrepancies are observed among the three types of seizure measurement methods: (C) 4-week seizure recall, (D–F) seizure diary, and (G–I) iCEM seizure detection. The greatest discrepancy occurred post-drug titration compared to pre-drug averages. Self-reported seizures increased while the iCEM-detected seizures decreased. Post-drug, there was also a change in the person recording the seizure diary, with the participant’s family taking on greater responsibility because the participant had a harder time recalling seizures. However, family members were not consistently available. This highlights reliability as an important consideration with either subjective or objective seizure detection. Also note, cyclical fluctuations in the iCEM long-term seizure average, suggesting a seizure cycle longer than three months. Conversely, (F and I) the longest inter-seizure interval showed no substantial increase (data gaps were excluded from this calculation), indicating treatment inefficacy. Despite this, the time required to conclusively determine the ineffectiveness of the new drug regimen remains unclear. QOLIE-89: Quality of Life in Epilepsy 89, iCEM implantable continuous EEG monitoring.

After the maximum dose was achieved, the longest inter-seizure interval did not increase more than threefold, and no period of seizure freedom was observed. No change in seizure type or focal to bilateral tonic–clonic seizures or seizure-related injuries were documented during the post-drug period. Following the drug change, the diary was maintained by a family member, as the participant was unable to record events due to seizure burden; only event counts were recorded, leaving it unclear whether focal to bilateral tonic–clonic seizures had truly decreased. Despite ongoing frequent seizures, perampanel was added three years later at the family’s request. This extended monitoring period highlights the challenges of making treatment decisions when clinical outcomes vary naturally over time and diary data, quality-of-life, or seizure severity information are missing and limited (see Fig. 1).

### 3.2 Projecting long-term trends in seizure rate for drug assessment

**Fig. 2:**
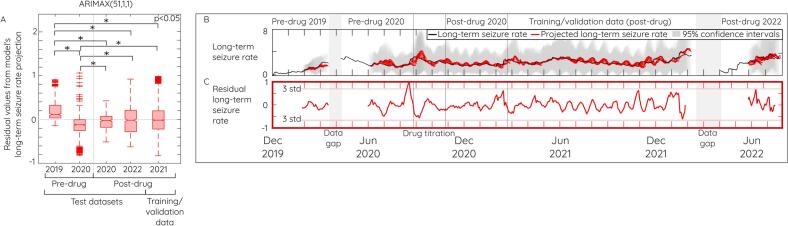
**Monitoring model performance using residual values may help to distinguish treatment (in)efficacy from seizure rate changes due to cycles.** (A) The ARIMAX(51,1,1) model, incorporating IED cycles, shows significant performance differences between pre- and post-drug data, indicating its ability to differentiate drug regimens. No significant differences were observed within the post-drug period. (B) Long-term (3-monthly) seizure rates and projected seizure rates with 95 % confidence intervals. (C) Residual values of the model or error in projecting seizure rates. Residuals within ± 3 standard deviations of the post-drug training/validation mean, indicate the model projected variations in seizure rate that was normal for the participant and these variations in seizure rate were unrelated to a change in medication. Positive residual values greater than the 3 standard deviations, that peaked in August 2020, signal temporary abnormal seizure increases and worse control, which could objectively support the decision to change medication. The changepoint, where residual values return to normal range, also provides and objective indicator that the long-term seizure rate will return to baseline. This information could enable earlier clinical decisions about treatment inefficacy, avoiding the need to extend the monitoring period for increased certainty — which was 3 years for this participant.

The final ARIMAX(51,1,1) model, incorporating IED cycles, outperformed models without cyclical information (see supplementary results). Mean squared errors was smaller when tested on data from the same drug regimen compared for data from a different regimen. Specifically, post-drug test datasets (2020 and 2022) were 0.17 and 0.13 seizures per day over 3-months^2^ compared to 0.49 and 0.58 for pre-drug test datasets (2019 and 2020). Model performance differed significantly across datasets (Kruskal Wallis test: H=418.77, df = 4, p = 2.44exp^-89^). However, post-hoc tests showed no difference between post-drug test datasets and the training/validation dataset (Fig. 2A), indicating consistent performance on unseen data from the same ASM regimen. However, significant differences between pre- and post-drug datasets were identified, indicating changes in model performance across drug regimens. See Fig. 2B–C for an example of how monitoring model performance can help distinguish drug-induced trends in seizure rate from normal variability, providing an individualised reference range and quantitative certainty to support clinical decision-making. For instance, if the seizure rate appears to decline after initiating a new drug regimen, but the model projects a return to the long-term average and model performance remains within the expected range (e.g., residuals within ±3 standard deviations), this could provide quantitative evidence that the drug is not having a meaningful effect and is likely ineffective—even within days of reaching the maximum tolerated dose.

When the model was applied to surrogate data, performance dropped, showing no significant difference between test datasets (supplementary Fig. 4 and supplementary Table 3). This suggests the model relies on temporal information from the timeseries and could not distinguish drug periods by random chance.

## 4 Discussion

This proof-of-concept case study provides preliminary evidence that a novel method – implantable continuous EEG monitoring (iCEM) combined with an individualised model to project long-term seizure rates – can differentiate between seizure rate and cycles of interictal epileptiform discharges recorded during different anti-seizure medication (ASM) regimens. With further development, this method has potential to provide quantitative feedback on ASM efficacy considerably earlier than current clinical approaches, thereby supporting clinical decision-making. Such advances could foreseeably improve treatment management for the majority of people with epilepsy, as 96 % rely on ASMs [28], and nearly two thirds will try multiple drug over several years [29] to achieve seizure control.

Reducing the time to find an optimal treatment, would have downstream impacts like reducing healthcare costs associated with ineffective treatments, lowering morbidity and mortality rates due to seizure related complications, decreased emergency department visits, hospital admissions, and days missed from school or work [30,31]. However, future research will need to validate this study using a model pre-trained on a cohort of patients to reduce training time requirements, which affects the time to treatment assessment.

Furthermore, this method was only investigated in the context of determining drug inefficacy, but it could also be used for assessing efficacy. This is based on evidence that interictal epileptiform discharges (IEDs) are a biomarker for ASM monitoring [32]. However, IEDs are not present in all people with epilepsy meaning this method will require alternate biomarkers and cycle detection methods for generalisability [32].

Nevertheless, our findings align with the few (case) studies showing objective seizure detection improves ASM efficacy evaluations [8,33]. However, simulations suggest only patients with very low self-reporting accuracy, like ours, benefit from objective monitoring to improve drug assessments [34]. Yet, self-reporting reliability probably fluctuates over time due to reasons like carelessness, fatigue, motivation, biases, or privacy concerns [35,36]. When seizure diaries can no longer be maintained, clinicians rely on average seizure recall, which can be inaccurate. Average seizure recall also misses vital information like the longest inter-seizure interval, which appears to be a more reliable measure of drug inefficacy for this case study. Recalling this inter-seizure interval could serve as a proxy, but its reliability is questionable. Although the diary and iCEM resulted in similar trends in the longest inter-seizure interval, trial participation might have biased results, highlighting the importance of objective monitoring. However, device adherence—such as keeping the device connected and charged—remains a challenge but is expected to improve with advancements in both device and service design.

The limitations of case studies and simulations make it unclear whether this method will improve ASM efficacy assessments. As the first study on long-term seizure projections for ASM monitoring, our findings should be viewed as methodological proof-of-concept evidence. Nonetheless, they support future exploration of this approach, given its potential impact if successful. Reassessment of the method in another retrospective study with less missing data (seizure frequency, seizure type, severity and quality of life data) will be necessary for a more accurate and clinically relevant evaluation. Building on the original retrospective evaluation, future work should test the method in real time, followed by assessment of reproducibility and generalisability.

Moreover, future studies could focus on optimising the projected long-term seizure average for clinical use. They could also refine seizure rate timescales to reduce variability, presumably due to circannual seizure cycles, and implement causal methods for cycle detection. While reducing drug assessment timeframes to under 1 year may be challenging, this method may improve on reports of 2–5 years of drug trial-and-error to achieve seizure control [34,38].

Additionally, studies could aim to improve automatic detection accuracy for seizures and IEDs [37]. Automatic, accurate discrimination of seizure types will also be crucial for evaluating treatment efficacy in patients with multiple seizure types, where differences in complication risk can influence treatment decisions. Lastly, refining the time horizon for projecting future seizure rates, and the time to wait before calculating residual values, could enhance clinical use, especially if clinical actions align with specific timesteps. However, lessons from seizure forecasting suggest challenges aligning performance metrics with clinical outcomes [13,39], necessitating further research.

## 5 Conclusion

Accurate long-term objective detection of seizures could improve the assessment of ASMs. However, simply calculating changes to seizure rates is insufficient when there is uncertainty regarding the appropriate duration to wait before evaluating therapy. Using a model based on an individual’s past seizure trends may help distinguish true drug-induced changes in seizure rate from natural fluctuations due to seizure cycles. Overall, we provide preliminary evidence that combining an implantable continuous EEG monitoring system with a personalised statistical models may offer a less biased approach for early and precise assessments of ASM efficacy, improving management and enhancing the quality of life for those living with epilepsy.

## CrediT authorship contribution statement

**A. Reynolds:** Writing – review & editing, Writing – original draft, Visualization, Validation, Methodology, Investigation, Formal analysis, Data curation, Conceptualization. **A. Lai:** Writing – review & editing, Supervision, Project administration, Funding acquisition, Data curation. **D.B. Grayden:** Writing – review & editing, Supervision. **M.J. Cook:** Writing – review & editing, Supervision, Resources. **A. Peterson:** Writing – review & editing, Supervision.

## Funding

This study did not receive any specific funding. However, the original study from which the data were collected received funding support from Epi-Minder Pty Ltd.

## Declaration of competing interest

The authors declare the following financial interests/personal relationships which may be considered as potential competing interests: Ashley Reynolds reports travel was provided by The University of Melbourne Graeme Clark Institute for Biomedical Engineering. Ashley Reynolds reports travel was provided by International Conference of Technology and Analysis of Seizures (ICTALS) 2025. Mark J. Cook reports a relationship with Epi-minder that includes: employment and equity or stocks. If there are other authors, they declare that they have no known competing financial interests or personal relationships that could have appeared to influence the work reported in this paper.
