# Supplementary material for: Early detection of antiseizure medication inefficacy using an implantable continuous EEG system and a personalized model: a case study
Source: Epilepsy Behav Rep. 2025 Sep 23;32:100829. doi: 10.1016/j.ebr.2025.100829 (PMC12510072; doi:10.1016/j.ebr.2025.100829)
Supplement: Supplementary Data 1 [file mmc1.docx]

Supplementary material

Method

Data Pre-Processing

To address gaps in the timeseries of seizures and interictal epileptiform discharges (IEDs), interpolation was implemented as per Baud et al. (2018) [1]. Interpolation between the means of the flanking segments of the gap, which were up to five times the duration of the gap, was performed. Gaussian white noise, with standard deviation matching that of the concatenated flanking segments, was added to the imputed values [1]. Hours of completely missing data that exceeded 20% of the maximum IEA cycle period were not interpolated to reduce error; instead data before and after the gap were analysed independently [1].

Feature Extraction

*Preliminary Feature Extraction*

Linear regression was undertaken between the variables: self-reported seizures per day, sub-scalp EEG seizure per day, and interictal discharge per day. This was conducted as a preliminary investigation into the possible use of these variables in a simple predictive linear regressor.

During the study trial, extensive COVID-19 lockdowns were enforced in Melbourne, Australia. The dates of these lockdowns were included as potential variables influencing seizure rates. Principal component analysis was also used to identify the top contributing independent variables affecting seizure rate variability.

*Long-Term Trend in Seizure Rate*

Seizure start times were converted into seizures per day, and a moving average over the past 3 months (90 days) was computed up to the current time point. A 3-month average was chosen to model the long-term trend in seizure rate because it is commonly used in both clinical and drug trial settings to evaluate the response to ASM [2]. A 3-month moving average also smooths out fluctuations in seizure rate, making it easier to observe trends at any point in time.

*Cycles of Interictal Epileptiform Discharges*

Since the wavelet transform can estimate the instantaneous phase of a continuous signal’s periodic components, IED cycles were estimated instead of seizure cycles. This is because, IEDs per hour were a continuous signal, unlike seizures per hour or day, which was a discrete signal, for this participant. IEDs per hour was used to identify all possible cycles between 1 day and 45 days, using the continuous Morlet wavelet transform [1]. Statistically significant cycle periods were identified using a permutation test. Two hundred random permutations of IEDs per hour were generated [3]. The continuous Morlet wavelet transform was applied to each permutation. A false discovery rate of 0.95 was used to set a threshold for the magnitude of the wavelet transform coefficients [3]. For each day, cycle periods with magnitudes above the threshold were considered statistically significant. Of these statistically significant periods, the period length with the greatest magnitude was taken as the daily dominant cycle period. For each day, the instantaneous phase of the dominant cycle period was computed. As per the study by Leguia et al. (2023) the sine and cosine of the phase were included as the exogenous variables [4] in the autoregressive model.

Model Selection

Here, we provide additional considerations, building on those described in the introduction, for employing an autoregressive model. Short-term seizure forecasting research has demonstrated that simple models, like generalised linear models and moving average models, can outperform complex machine learning models while reducing training data requirements [4, 5]. Autoregressive models, like these simpler models, also avoid issues associated with black-box models, which are important for clinical acceptance [6]. Furthermore, personalised models that use patient specific features tend to outperform generalised ones [4, 7, 8]. Therefore, models used in this study included autoregressive models (AR), moving average models (MA), autoregressive integrated moving average (ARIMA) models and autoregressive integrated moving average models with exogenous variables (ARIMAX).

Model Parameterisation

To reduce the time to assess ASM, the amount of model input data to make a single projection had to be as short as possible. Therefore, the duration of input data depended on the lag included in the model, so for an AR(50) model, input data was 51 days duration. For all models, a long-term seizure rate was projected 14-days into the future, termed the time horizon, which was generated and updated daily. Fourteen days was used to capture possible changes to seizure rate when ASMs are slowly titrated, allowing for the new ASM to reach steady state and the old ASM to be eliminated.

Significant lags for autoregressive models were identified using the partial autocorrelation function with 5% significance limits. Hyperparameters such as autoregressive lag order (0 to 90), differencing order (0 to 2), and moving average order (0 to 90) were optimised using a grid-search and repeated hold-out validation with a 70% and 30% split. This method repeatedly sampled the training/validation split point 100 times [9]. The maximum 90-day lag order was selected as seizure rate was already averaged over 90 days. The average mean squared error was used as a standard performance metric to assess and select the final model. The Ljung-Box test was applied to check for residual autocorrelation, and Engle's ARCH test was used to detect autoregressive conditional heteroscedasticity effects in the residuals. Both tests used a significance level of α=0.05.

Statistics

*Evaluating model performance*

An autoregressive model (AR(1)) and moving average (MA(1)) model, both with lags of 1 day, were considered naïve models to benchmark model performance. The models were evaluated based on the mean squared error from the validation dataset. The final model and best performing autoregressive and moving average models were then evaluated on the four held-out test datasets. Model performance across the test datasets were assessed using the Kruskal-Wallis test (α=0.05) with Wilcoxon Rank Sum for post-hoc comparisons to identify significant differences in residual values. For a model to differentiate drug treatments, it should perform similarly on test datasets from the same drug regimen as the training/validation data. However, performance should vary when the test datasets come from a different regimen. Specifically, both pre-drug test datasets should be significantly different from the post-drug training/validation datasets, but there should be no difference between the test datasets from the same drug period.

*Surrogate timeseries*

To assess whether the models' results were due to random chance, they were tested on a surrogate timeseries [7]. These surrogates, generated by randomly shuffling each timeseries (seizure per day and IED per hr) 1000 times, disrupted temporal relationships to create a null hypothesis dataset. The surrogate time series were divided into training, validation, and test datasets as described in the methods in the main text. The final model, trained on the original time series, was then applied to the surrogate test datasets. Residual values were compared using the Kruskal-Wallis test to identify significant differences.

Results

Preliminary Feature Extraction

Principle components analysis found quality of life scores, seizure severity scores or COVID-19 lockdowns contributed to less than 5% of the variability in seizure rate and were not considered as exogenous variables, for a parsimonious model. There were weak correlations with poorly fitting linear models between seizure rate (4 week recall, diary reported or device detected) and quality of life scores and seizure severity (Supplementary Figure 1). This is likely related to the few data points and missing time points for each survey. Furthermore, there were only weak linear correlations between rates of self-reported seizures, sub-scalp EEG seizures, or interictal epileptiform activity (Supplementary Table 1).


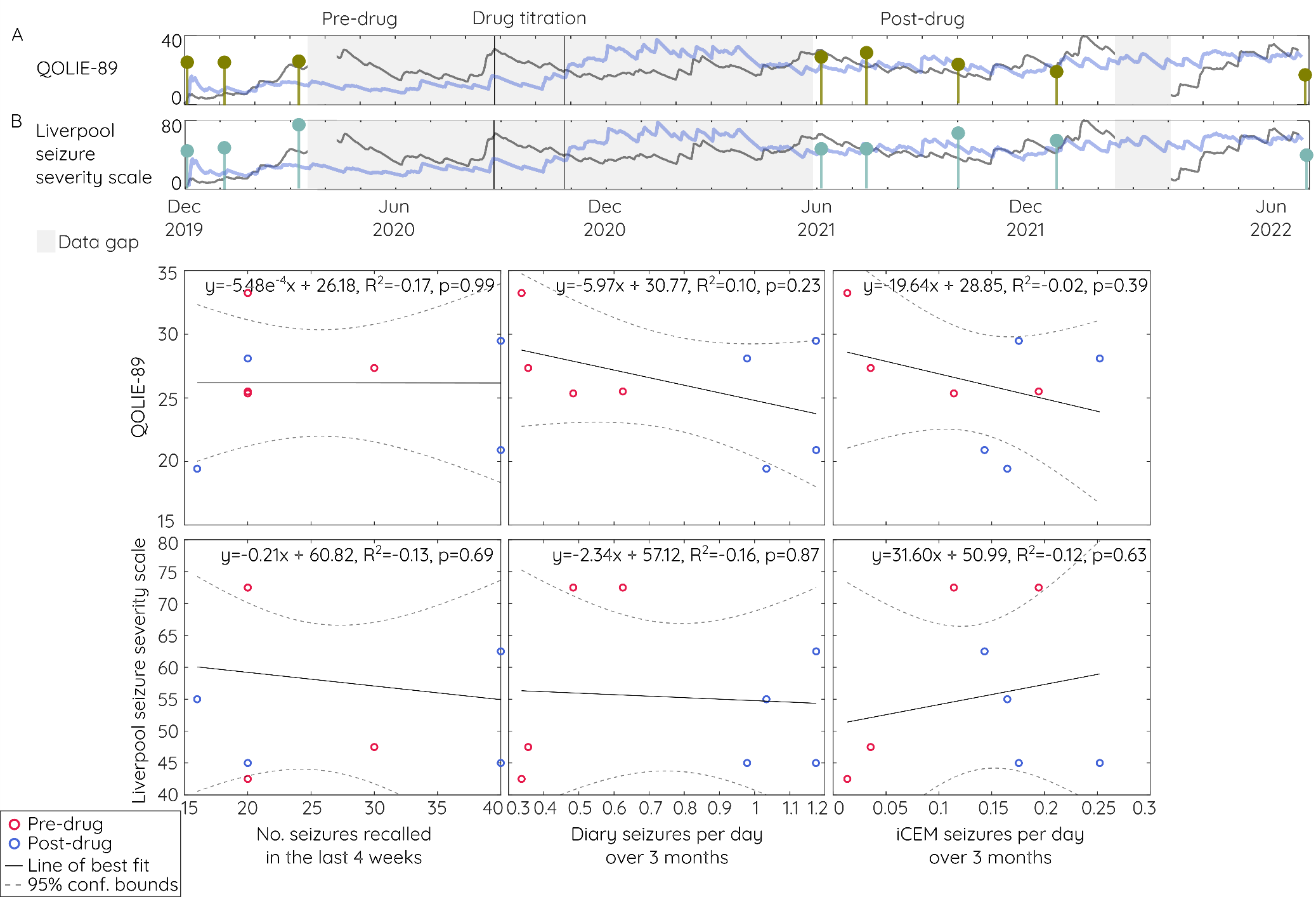


**Supplementary Figure 1. Correlations between seizure rate (4-week recall, diary, and iCEM-detected seizures) and both quality-of-life scores and seizure severity scale scores.**

**Supplementary Table 1. Feature selection process, linear regression to exclude weakly correlated variables from the final model.** Linear regression between the variables: daily rates of interictal epileptiform discharges, seizures reported in a diary and seizures detected on sub-scalp EEG. These variables were weakly correlated for all the different anti-seizure medication treatment periods. Therefore, interictal epileptiform discharges per day, diary seizures per day and sub-scalp EEG seizures per day were not included as variables in the autoregressive models. DF: Degrees of freedom.

| **Drug period** | **F** | **df** | **p-value** | **R2 adjusted** | **Intercept** | **95% confidence interval** | | **p-value** | **Coefficient estimate** | **95% confidence interval** | | **p-value** |
| --- | --- | --- | --- | --- | --- | --- | --- | --- | --- | --- | --- | --- |
| **Interictal epileptiform discharges per day to predict diary seizures per day** | | | | | | | | | | | | |
| **Pre-drug 2019** | 6.48 | 1, 99 | 0.01 | 0.05 | 0.19 | -0.11 | 0.49 | 0.21 | 2.10e-4 | 4.63e^-5^ | 3.74 e^-4^ | 0.01 |
| **Pre-drug 2020** | 27.45 | 1, 134 | 6.11e^-7^ | 0.16 | -0.26 | -0.66 | 0.13 | 0.19 | 5.91e^-4^ | 3.68e^-4^ | 8.14e^-4^ | 6.11e^-7^ |
| **Titration 2020** | 1.53 | 1, 59 | 0.22 | 8.71e^-3^ | 0.21 | -0.52 | 0.95 | 0.57 | 3.04e^-4^ | -1.90 e^-4^ | 7.98e^-4^ | 0.22 |
| **Post-drug 2020** | 21.62 | 1, 98 | 1.04e^-5^ | 0.17 | -0.42 | -1.49 | 0.65 | 0.44 | 1.61e^-3^ | 9.25e^-4^ | 2.30e^-3^ | 1.04e^-5^ |
| **Post-drug 2022** | 10.41 | 1, 116 | 1.63e^-^3 | 0.07 | 0.59 | -0.09 | 1.28 | 0.09 | 8.99e^-4^ | 3.47e^-4^ | 1.45 e^-3^ | 1.63 e^-3^ |
| **Post-drug training/ validation** | 28.53 | 1, 367 | 1.62e^-7^ | 0.07 | 0.24 | -0.17 | 0.65 | 0.25 | 7.20e^-4^ | 4.55e^-4^ | 9.85 e^-4^ | 1.62e^-7^ |
| **Interictal epileptiform discharges per day to predict sub-scalp EEG seizures per day** | | | | | | | | | | | | |
| **Pre-drug 2019** | 48.53 | 1, 99 | 3.63e^-10^ | 0.32 | -0.80 | -2.06 | 0.47 | 0.21 | 2.44e^-3^ | 1.74 e^-3^ | 3.13 e^-3^ | 3.63e^-10^ |
| **Pre-drug 2020** | 46.03 | 1, 134 | 3.42e^-10^ | 0.25 | -0.75 | -2.43 | 0.93 | 0.38 | 3.24e^-3^ | 2.30 e^-3^ | 4.18 e^-3^ | 3.42e^-10^ |
| **Titration 2020** | 42.52 | 1, 59 | 1.74e^-8^ | 0.41 | -1.03 | -2.56 | 0.50 | 0.18 | 3.34e^-3^ | 2.32 e^-3^ | 4.37 e^-3^ | 1.74e^-8^ |
| **Post-drug 2020** | 12.81 | 1, 98 | 5.39e^-4^ | 0.11 | 0.46 | -1.57 | 2.48 | 0.66 | 2.34e^-3^ | 1.05e^-3^ | 3.65 e^-3^ | 5.39e^-4^ |
| **Post-drug 2022** | 28.14 | 1, 116 | 5.47e^-7^ | 0.19 | -1.56 | -4.78 | 1.65 | 0.34 | 6.93e^-3^ | 4.34e^-3^ | 9.52e^-3^ | 5.47e^-7^ |
| **Post-drug training/ validation** | 77.45 | 1, 367 | 5.43e^-17^ | 0.17 | -0.23 | -1.54 | 1.08 | 0.73 | 3.80e^-3^ | 2.95e^-3^ | 4.65e^-3^ | 5.43e^-17^ |
| **Sub-scalp EEG seizures per day to predict diary seizures per day** | | | | | | | | | | | | |
| **Pre-drug 2019** | 4.07 | 1, 99 | 0.05 | 0.03 | 0.39 | 0.19 | 0.60 | 0.00 | 0.04 | 6.40e-4 | 0.08 | 0.05 |
| **Pre-drug 2020** | 65.03 | 1, 134 | 3.66e^-13^ | 0.32 | 0.09 | -0.16 | 0.33 | 0.48 | 0.13 | 0.10 | 0.16 | 3.66e^-13^ |
| **Titration 2020** | 3.14 | 1, 59 | 0.08 | 0.03 | 0.33 | -0.17 | 0.83 | 0.19 | 0.08 | -0.01 | 0.18 | 0.08 |
| **Post-drug 2020** | 26.34 | 1, 98 | 1.45e^-6^ | 0.20 | 0.83 | 0.18 | 1.47 | 0.01 | 0.25 | 0.16 | 0.35 | 1.45e^-6^ |
| **Post-drug 2022** | 19.29 | 1, 116 | 2.49e^-5^ | 0.14 | 1.09 | 0.67 | 1.52 | 1.20e^-6^ | 0.08 | 0.04 | 0.11 | 2.49e^-5^ |
| **Post-drug training/ validation** | 47.80 | 1, 367 | 2.11e^-11^ | 0.11 | 0.69 | 0.44 | 0.95 | 2.39e^-7^ | 0.10 | 0.07 | 0.13 | 2.11e^-11^ |

Model selection

The ARIMAX(51,1,1) model described by equation 1 was selected as the final model because it had the smallest average mean squared error compared to the naïve models (Supplementary Table 2 reports the mean squared error of the residual values from the top 10 models). See Supplementary Figure 2 for the variables included in the model. As mentioned in the introduction, an autoregressive model uses a linear combination of past data to model and project future values. An ARIMAX model – short for autoregressive integrated moving average model with exogenous variables – builds on this by incorporating three additional components [10]. First, it is integrated, meaning it smooths data to remove trends. Second, it includes a moving average component to account for previous forecast error to improve accuracy. Third, it contains exogenous variables, which are external factors that influence the data being modelled[10]. In this case, the phase of the IED cycle served as the exogenous variable.

($1-\emptyset_{1}L-... -\emptyset_{51}L^{51})(1-L)y_{t}=c+X_{1}\beta_{1}+ X_{2}\beta_{2}+(1+\theta_{1}L)\varepsilon_{t}$ (1)

Equation 1 describes the final model, ARIMAX(51,1,1), where $\emptyset_{n}$ represents the coefficient of the autoregressive lag $L^{n}$, with lag orders, $n$ ranging from 1 day to 51 days of the long-term seizure rate $y_{t}$. The constant term is $c$. $X_{1}$ is the coefficient of cosine of the phase of the IED cycle $\beta_{1}$, and $X_{2}$ is the coefficient of the sine of the phase of the IED cycle $\beta_{2}$. $\varepsilon_{t}$ is modelled as a series of independent and identically distributed Gaussian random variables.

For this same model, the Ljung-box test was negative for residual autocorrelation ($H$=10.26, p=1.00) and the Engle’s ARCH test indicated no autoregressive conditional heteroscedastic effects of residual values ($H$=38.20, p=0.91).

**Supplementary Table 2. Performance of the top 10 potential models.** The ARIMAX([1:51],1,1) with the interictal epileptiform discharge (IED) cycle had the lowest mean squared error and was selected as the final model along with the AR(1) and MA(1:50) to test. AR: autoregressive, MA: moving average, ARIMA: autoregressive integrated moving average, ARIMAX: autoregressive integrated moving average with exogenous factors.

| **Model** | **Mean squared error of residual values** |
| --- | --- |
| AR(1) | 2.27exp^-4^ |
| MA(1) | 1.03exp^-3^ |
| MA(1:50) | 3.22exp^-4^ |
| ARIMAX([1:51],1,1) + IED cycle | 2.06exp^-4^ |
| ARIMAX([1:2,51],1,1) + IED cycle | 3.15exp^-4^ |
| ARIMAX([1:2,34,51],1,1) + IED cycle | 3.17exp^-4^ |
| ARIMAX([1:55],1,1) + IED cycle | 2.26exp^-4^ |
| ARIMAX([1:4,55],1,1) + IED cycle | 2.72exp^-4^ |
| ARIMAX([1,2,55],1,1) + IED cycle | 3.19exp^-4^ |
| ARIMAX(1:20,1,1) + IED cycle | 2.21exp^-4^ |

***
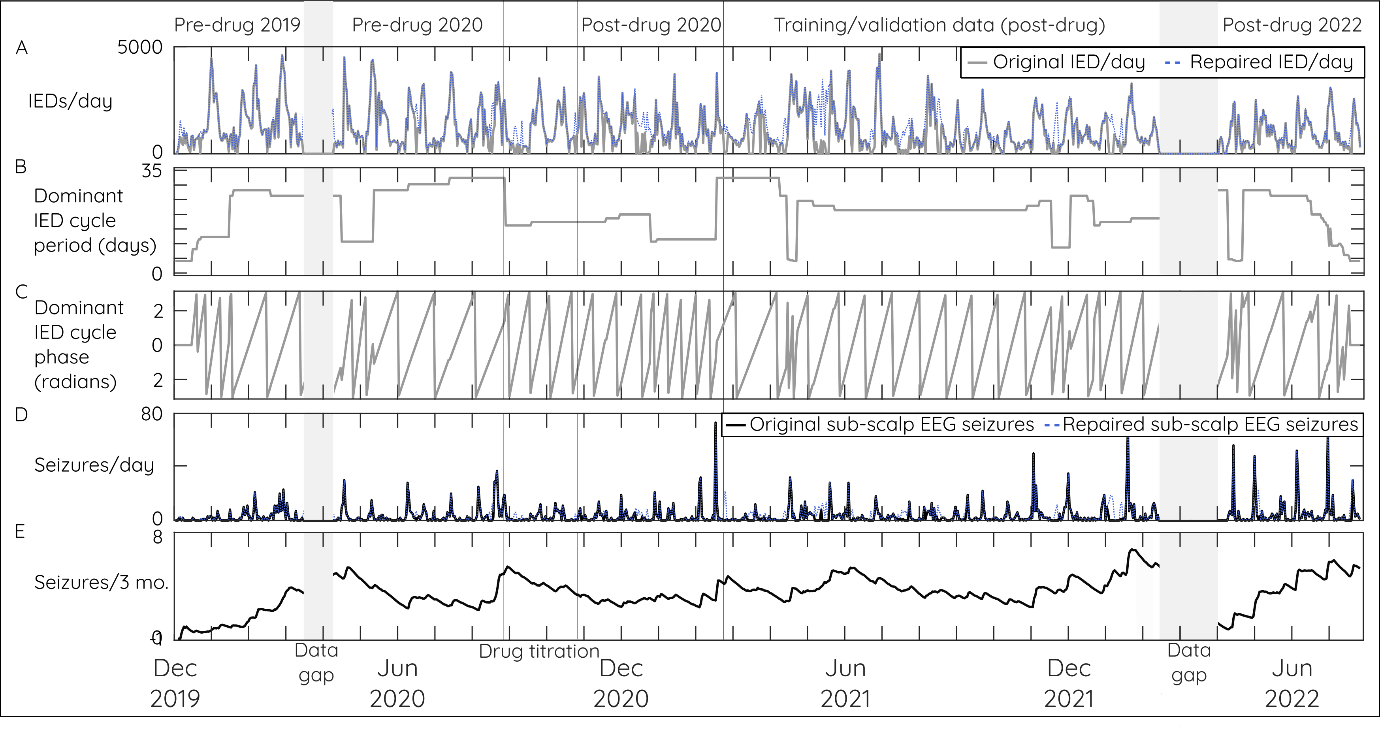
***

**Supplementary Figure 2. Variables included in the final model.** (A) Interictal epileptiform discharges (IEDs) per day were used to extract (B) the dominant IED cycle period and (C) phase, which was included in (E) the long-term (3-monthly) seizure rate in the model.

Model performance

The autoregressive AR(1) and moving average MA(50) models were too simple, leading to variable performance across all test datasets regardless of drug regimen (Supplementary Figure 3).


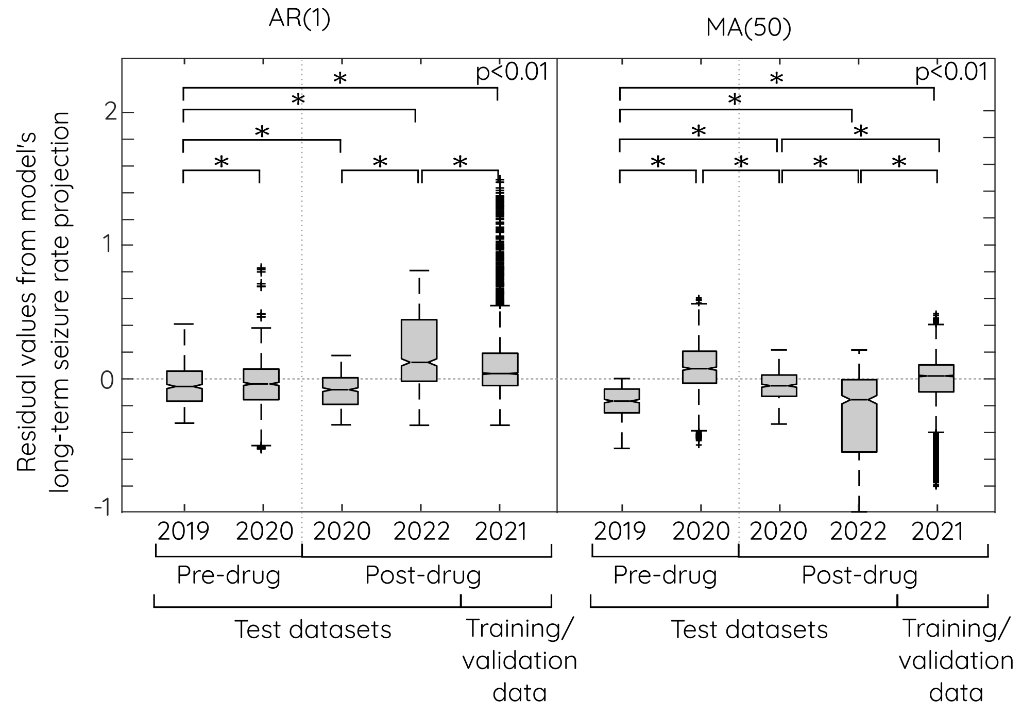


**Supplementary Figure 3. Simple models show varying performance across all test datasets.** The naïve autoregressive AR(1) model and the moving average model that had the smallest mean squared error (MA(50)) were tested on four datasets. Two test datasets were from each drug regimen. For the model to effectively differentiate between drug periods, significant differences in performance are expected between tests datasets from pre-drug and post-drug periods. However, both models show performance differences between the pre-drug 2019 data and all post-drug data but not between pre-drug 2020 and the post-drug data. Moreover, significant differences in model performance should not be observed between test datasets within the same drug period (e.g., post-drug 2020 and post-drug 2022) or between these post-drug test datasets and the training/validation data (post-drug 2021). However, this was not the case for these two models.

For the final model (results in Figure 2 of the main text), there was a statistically significant performance difference between the two pre-drug test datasets, potentially indicating a temporal effect. Specifically, November 2019-March 2020 may have significantly differed from the training/validation data (February 2021-February 2022), whereas data from April-August 2020 may have been more similar to the training/validation period. Other possible factors contributing to these differences include recency of implantation, variable device usage from novice to experienced, the development of drug tolerance, other changes in drug metabolism, drug-drug interactions and the development or exacerbation of comorbidities.


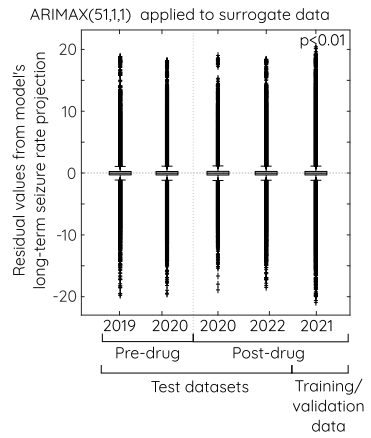


**Supplementary Figure 4. The ARIMAX(51,1,1)** **model’s ability to differentiate drug regimens depends on IED cycle information.** The trained ARIMAX(51,1,1) model, incorporating IED cycle information, performs poorly across all surrogate datasets. This is due to random shuffling of timeseries, which destroys useful temporal patterns. This is evidenced by the large residual values and lack of statistically significant performance differences between any of the datasets.

**Supplementary Table 3. The error in the models across the four test datasets from different drug regimens.**

|  | **Training/validation (post-drug)** | | **Pre-drug 2019** | | **Pre-drug 2020** | | **Post-drug 2020** | | **Post-drug 2022** | | **Kruskal-wallis test H** | **p-value** |
| --- | --- | --- | --- | --- | --- | --- | --- | --- | --- | --- | --- | --- |
|  | **Mean squared error** | **Residuals** | **Mean squared error** | **Residuals** | **Mean squared error** | **Residuals** | **Mean squared error** | **Residuals** | **Mean squared error** | **Residuals** |  |  |
| AR(1) | 2.27e^-4^ | M=8.63e-2, SD=2.53e-1, 95% CI [9.40e-2, 9.40e-2] | 3.21e-1 | M=-2.68e-03, SD=1.83e-02, 95% CI  [-1.05e-03,  -1.05e-03] | 3.21e-1 | M=-5.62e-2, SD=1.83e-1, 95% CI  [-4.48e-2,  -4.48e-2] | 1.15e-1 | M=-8.97e-2, SD=1.26e-1, 95% CI  [-7.83e-2,  -7.83e-2] | 7.90e-1 | M=2.01e-1, SD=3.06e-1, 95% CI [2.23e-1, 2.23e-1] | 376.24 | 3.78e-80 |
| MA(50) | 3.22e^-4^ | M=8.63e-2, SD=2.53e-1, 95% CI [9.40e-2, 9.40e-2] | 1.68e-1 | M=-2.68e-03, SD=1.83e-02, 95% CI  [-1.05e-03,  -1.05e-03] | 3.58e-1 | M=-5.62e-2, SD=1.83e-1, 95% CI  [-4.48e-2,  -4.48e-2] | 1.14e-1 | M=-8.97e-2, SD=1.26e-1, 95% CI  [-7.83e-2,  -7.83e-2] | 9.73e-1 | M=2.01e-1, SD=3.06e-1, 95% CI [2.23e-1, 2.23e-1] | 807.08 | 3.41e-172 |
| ARIMAX(51,1,1) | 2.06e^-4^ | M=-2.86e-3, SD=3.39e-1, 95% CI [7.35e-3, 7.35e-3] | 5.10e-1 | M=2.00e-02, SD=2.52e-02, 95% CI [2.22e-02, 2.22e-02] | 5.00e-1 | M=-9.11e-2, SD=2.08e-1, 95% CI  [-7.80e-2,  -7.80e-2] | 1.90e-1 | M=-5.10e-2, SD=1.95e-1, 95% CI  [-3.34e-2,  -3.34e-2] | 1.80e-1 | M=-1.24e-1, SD=3.10e-1, 95% CI [-1.47e-1,  -1.27e-1] | 418.77 | 2.44e-89 |
| ARIMAX  (51,1,1) with surrogate data | 3.90e-1 | M=-3.66e-2,  SD=1.05, 95% CI  -4.65e-2,  -2.66e-2] | 3.90e-1 | M=-3.64e-03, SD=1.06e+00, 95% CI  [-9.61e-03,  -3.60e-03] | 3.90e-1 | MD=-2.36e-2, SD=1.07, 95% CI [-9.49e-2,  -2.24e-2] | 3.90e-1 | M=-2.35e-2, SD=1.05, 95% CI  [-5.34e-2,  6.31e-3] | 3.90e-1 | M=-1.55e-2, SD=1.07, 95% CI  [-4.02e-2, 9.15e-3] | 116.32 | 0.69 |

## References

[1] M. O. Baud *et al.*, "Multi-day rhythms modulate seizure risk in epilepsy," *Nature communications,* vol. 9, no. 1, pp. 1-10, 2018.

[2] E. Ben-Menachem, J. W. Sander, M. Privitera, and F. Gilliam, "Measuring outcomes of treatment with antiepileptic drugs in clinical trials," *Epilepsy & Behavior,* vol. 18, no. 1, pp. 24-30, 2010/05/01/ 2010, doi: <https://doi.org/10.1016/j.yebeh.2010.04.001>.

[3] C. Friedrichs‐Maeder, T. Proix, T. K. Tcheng, T. Skarpaas, V. R. Rao, and M. O. Baud, "Seizure Cycles under Pharmacotherapy," *Annals of neurology,* 2024.

[4] M. G. Leguia *et al.*, "Learning to generalize seizure forecasts," *Epilepsia,* vol. 64, pp. S99-S113, 2023.

[5] D. M. Goldenholz, C. Eccleston, R. Moss, and M. B. Westover, "Prospective validation of a seizure diary forecasting falls short," *Epilepsia,* 2024.

[6] B. Abbasi and D. M. Goldenholz, "Machine learning applications in epilepsy," *Epilepsia,* vol. 60, no. 10, pp. 2037-2047, 2019.

[7] T. Proix *et al.*, "Forecasting seizure risk in adults with focal epilepsy: a development and validation study," *The Lancet Neurology,* vol. 20, no. 2, pp. 127-135, 2021, doi: 10.1016/s1474-4422(20)30396-3.

[8] R. E. Stirling *et al.*, "Seizure Forecasting Using a Novel Sub-Scalp Ultra-Long Term EEG Monitoring System," *Front Neurol,* vol. 12, p. 713794, 2021, doi: 10.3389/fneur.2021.713794.

[9] V. Cerqueira, L. Torgo, and I. Mozetič, "Evaluating time series forecasting models: an empirical study on performance estimation methods," *Machine Learning,* vol. 109, no. 11, pp. 1997-2028, 2020, doi: 10.1007/s10994-020-05910-7.

[10] R. Hyndman, *Forecasting: principles and practice*. OTexts, 2018.
